# Just-In-Time Adaptive Interventions to Promote Behavioral Health: Protocol for a Systematic Review

**DOI:** 10.2196/58917

**Published:** 2025-02-11

**Authors:** Lauren M Henry, Morkeh Blay-Tofey, Clara E Haeffner, Cassandra N Raymond, Elizabeth Tandilashvili, Nancy Terry, Miryam Kiderman, Olivia Metcalf, Melissa A Brotman, Silvia Lopez-Guzman

**Affiliations:** 1 Emotion and Development Branch National Institute of Mental Health Bethesda, MD United States; 2 National Institutes of Health Library Office of Research Services, Office of the Director National Institutes of Health Bethesda, MD United States; 3 Phoenix Australia Department of Psychiatry The University of Melbourne Melbourne Australia

**Keywords:** just-in-time adaptive interventions, JITAI, behavioral health, systematic review, behavior change, health outcomes, accessibility, digital treatment delivery, mobile phone

## Abstract

**Background:**

The goal of just-in-time adaptive interventions (JITAIs) is to use mobile, digital tools to provide individuals with personalized interventions at the optimal time and in the optimal context. Accordingly, JITAIs are promising for advancing accessible, equitable, and evidence-based treatment for behavioral health. To guide future inquiry in this space, a review of the literature is needed to describe the state of research on JITAIs for behavioral health.

**Objective:**

This study aims to systematically review the literature to describe the landscape of existing JITAIs for behavioral health at any stage of intervention development. In addition, conditional upon a sufficiently homogeneous literature, we will conduct meta-analyses to investigate the effectiveness of JITAIs for promoting distal outcomes (here, aspects of behavioral health) and proximal outcomes (eg, emotion regulation).

**Methods:**

This systematic review is being conducted in accordance with the PRISMA-P (Preferred Reporting Items for Systematic Reviews and Meta-Analyses Protocols). We developed our search strategy and executed the literature search in collaboration with biomedical librarians; 5 databases (PubMed, Embase, Cochrane Library, Web of Science: Core Collection, and APA PsycINFO) were searched, and results were managed using EndNote 20 (Clarivate). We are screening (title, abstract, and full text) all records in duplicate in Covidence according to eligibility criteria. Data items will be extracted, and risk of bias will be assessed in duplicate from the included articles in Covidence. We will summarize JITAI characteristics in tables and text. We will conduct meta-analyses for the distal and proximal outcomes conditional upon sufficient homogeneity in subgroups. Moderation (conditional upon sufficient heterogeneity of outcomes) and mediation (ie, whether changes in proximal outcomes mediate the relation between JITAIs and distal outcomes) will be conducted as appropriate. We will investigate publication bias and use the Grading of Recommendations Assessment, Development and Evaluation to characterize the quality of evidence of our estimates.

**Results:**

The search strategy was developed between July 2023 and November 2023. The literature search was executed between November 2023 and December 2023. Title and abstract screening began in December 2023, and full-text screening began in May 2024. Data extraction and analyses have not begun.

**Conclusions:**

Here, we propose a systematic review to assess the state of the literature on JITAIs for behavioral health. The insights derived from this study will describe the literature on JITAIs in promoting behavioral health, reinforce JITAI definitions, clarify JITAI elements, and inform the next steps in JITAI research.

**International Registered Report Identifier (IRRID):**

PRR1-10.2196/58917

## Introduction

### Background

Mental illness has devastating consequences for individuals, their families, and society. Those in need of treatment are woefully underserved, with a dearth of professionals relative to patients [1]. Race and ethnicity, geography, and socioeconomic status influence who can and who cannot access care [2]. For the subset of individuals who receive treatment, lack of evidence-based care [3] and issues with dropout [4] and nonresponse [5,6] limit recovery. New solutions are critical for reaching and equitably and effectively treating people suffering from mental illness. Technological advances may provide a pathway to more equitable evidence-based care at scale. While structural barriers (eg, cost and transportation) impede access to in-person health care, mobile devices are plentiful and may allow for greater population coverage [7,8]. Furthermore, while treatment may be perceived as stigmatizing, further reducing service use [9,10], research provides preliminary support for willingness to initiate and maintain engagement with digital interventions (even in severe mental illness) [11]. Beyond accessibility and acceptability, treatment delivered through technology allows for the provision of therapeutic support when and where it is most needed—outside of the clinic and within patients’ daily lives [12,13]. After a prescribed treatment is delivered, technological solutions can be seamlessly integrated and accessed again to boost skills and maintain treatment gains [12,13].

### Just-In-Time Adaptive Interventions

Despite the potential benefits of digital treatment delivery, evidence for the effectiveness of intervening using standalone mobile applications is limited [14]. Still, just-in-time adaptive interventions (JITAIs) are promising [15-18]. The goal of JITAIs is to target mechanisms for therapeutic change at the time and in the context that is optimal for the individual [19,20]. JITAIs have the potential to capitalize on states of vulnerability and opportunity [20]. That is, JITAIs can intervene when individuals are susceptible to negative or positive change. For example, capitalizing on a state of opportunity might be prompting an adult with social anxiety disorder to approach others while in a public place. JITAIs may be able to intervene when individuals are maximally receptive or most likely (willing and able) to accept a specific intervention [20]. To do so, JITAIs leverage real-time data collected passively or actively from smartphones, including or excluding paired wearable devices in combination with personalized algorithms [19-21].

JITAIs are defined by six elements: (1) distal outcome (long-term goal of the JITAI), (2) proximal outcome (short-term goal of the JITAI, which may be a mediator of the distal outcome), (3) tailoring variable (baseline or time-varying information on the individual that informs which interventions to deploy at which decision points), (4) decision point (time frame when an intervention option is, or is not, deployed), (5) decision rule (operationalization of which intervention option should be used, when, and for whom), and (6) intervention options (set of potential components that may be deployed toward behavior change at a given decision point). JITAIs are being developed for a variety of problems, including substance use [18,22], affective disorders [23], and stress management [24]. The potential impact of an intervention (here, a JITAI) is dependent on the potency of its mechanistic target. Some, but not all, JITAIs that aim to augment behavioral health outcomes describe doing so through purported proximal outcomes [23]. Importantly, to increase the likelihood that JITAIs will be effective, their development must be informed by well-established findings from psychiatric research. For example, deficits in emotion regulation, or efforts to modify the intensity or duration of an emotion, are a transdiagnostic pathway in the development and maintenance of psychopathology [25,26]. Importantly, emotion regulation is malleable in that it can be augmented with intervention [27]. As such, emotion regulation is a key exemplar candidate transdiagnostic treatment target for behavioral health. Emotion regulation skills are aspects of empirically supported treatments for psychopathology [28,29] and have been shown to mediate and moderate the effects of interventions on outcomes [27]. Increasingly, emotion regulation skills have been incorporated into digital health interventions [30-33].

There are 2 major gaps in understanding the potential use of JITAIs in behavioral health care [34]. Henceforth, we use the term “behavioral health” to refer to psychological disorders and symptoms (including substance use disorders), as well as physical symptoms related to life stressors and crises. First, little research has summarized the effectiveness of JITAIs for behavioral health conditions. Intervention development is a multistage process that involves iteratively tailoring a program through repeated evaluation and from accumulating evidence [35-37]. For example, the National Institutes of Health’s Obesity-Related Behavioral Intervention Trials (ORBIT) model provides a framework for intervention development that starts with establishing a significant clinical question and progresses through design (ie, defining, refining), preliminary testing (eg, proof-of-concept, feasibility pilots), efficacy trials, and finally effectiveness research. At any stage, in the case of a suboptimal outcome, the model would dictate that the investigator returns to an earlier phase for intervention refinement. All considered, the development of an intervention can take over 10 years before the program settles into its finalized form. What’s more, by design, a finalized form of an intervention may never be achieved. Interventions must be optimized to strike a strategic balance between effectiveness, affordability, scalability, and efficiency. That strategic balance may vary across time and context [36]. Frameworks like multiphase optimization strategy (MOST) can be used to guide these optimization efforts. Experimental designs should be matched to the research question and stage of development (eg, microrandomized trial [38] or sequential multiple assignment randomized trial [SMART] [39] during optimization, randomized controlled trial during evaluation [35]). JITAIs as intervention frameworks were conceptualized 10 years ago [19]. Considering the intervention development process, the majority of published JITAIs likely either do not exist in their finalized form or are constantly evolving through optimization. Still, systematic reviews and meta-analyses exist that have examined the impact of JITAIs on the promotion of physical health, providing some early support for JITAIs as a digital health tool [15,16]. The extant systematic reviews relevant to behavioral health have evaluated JITAIs either within a larger health promotion framework or for a specific psychiatric disorder. In their meta-analysis of JITAIs for physical and mental health (eg, healthy diet, weight loss, diabetic management, addiction, bipolar disorder, and anxiety), Wang and Miller [17] found a large effect of JITAIs relative to a waitlist control (Hedges' *g*=1.653). In their systematic review of JITAIs for substance use, Perski et al [18] found mixed results. Second, few systematic reviews [23] have described JITAI proximal outcomes despite the important role that proximal outcomes play in elucidating intervention mechanisms of action [40]. Here, we propose a systematic review (and meta-analysis, as appropriate) of JITAIs targeting proximal outcomes (eg, emotion regulation) to improve behavioral health outcomes.

### Objective

The objective of this review is to describe the state of the literature on JITAIs for behavioral health outcomes. Given that JITAIs originated in the past 10 years, our primary methodological approach for addressing our objective will be qualitative. That is, through a systematic review of the literature, we will extract, report, and describe the 6 elements (ie, distal outcome, proximal outcome, tailoring variable, decision point, decision rule, and intervention options) for each JITAI, regardless of the stage of intervention development (eg, preliminary testing, feasibility, clinical trial). In doing so, we will reinforce the existing JITAI framework [19,20] and provide a roadmap for the development of future JITAIs to improve behavioral health. Our secondary methodological approach for addressing our objective will be quantitative. That is, if it is determined that our included studies are sufficiently homogeneous, we will conduct meta-analyses to examine the effectiveness of JITAIs in promoting (1) aspects of behavioral health as distal outcomes and (2) proximal outcomes. We will examine moderators (eg, sociodemographic variables, symptom severity) conditional upon sufficient heterogeneity in outcomes. Given the importance of developing JITAIs incorporating empirically supported intervention targets, we will focus our discussion on emotion regulation as one proximal outcome of interest.

## Methods

### Overview

We used the PRISMA-P (Preferred Reporting Items for Systematic Reviews and Meta-Analyses Protocols) checklist to write this systematic review protocol [41]. Multimedia Appendix 1 shows the PRISMA-P checklist.

### Eligibility Criteria

Textbox 1 shows the inclusion and exclusion criteria. After examining a subset of records, it became clear that we were unable to determine with certainty if interventions were JITAIs (ie, just-in-time and adaptive) and if behavioral health outcomes were reported by examining article titles and abstracts. As such, to prevent excluding relevant articles during title and abstract screening, we retained a subset of our inclusion and exclusion criteria for this stage. That is, we focused on identifying articles using digital methods and proposing specific interventions and articles of the correct format (eg, excluding reviews and case reports). The full eligibility criteria are being used for full-text screening.

**Eligibility criteria for systematic review.**

Inclusion criteria:

- Population: All individuals, regardless of age, sex and gender, race and ethnicity, socioeconomic status, geographic location, and other aspects of identity.
- Intervention design: Just-in-time adaptive interventions (JITAIs), that is:
  Just-in-time: deployed when the individual needs it.
  Adaptive: incorporates time-varying or contextual or both types of information for individualization, leveraged through passively or actively collected data from smartphones or wearable devices.
  Intervention: activities intended to modify behavior, thoughts, or emotions.
- Outcome: Behavioral health.
- Article format: All languages; empirical research studies; publication in a peer-reviewed journal.

Exclusion criteria:

- Population: None.
- Intervention design: No JITAI.
- Outcome: Physical health and wellness outcomes that do not directly focus on behavioral health.
- Article format: Reviews, meta-analyses, case reports, dissertations, theses, and conference abstracts.

### Information Sources

A total of 5 databases were searched by a biomedical librarian (NT): PubMed/MEDLINE (US National Library of Medicine), Embase (Elsevier), Cochrane Library (John Wiley & Sons), Web of Science: Core Collection (Clarivate), APA PsycINFO (Dialog and Clarivate). To identify relevant articles missed by the search strategy, reviewers (LMH, MB-T, CEH, CNR, ET) will scan the bibliographies of included studies and relevant review articles. Protocols, feasibility studies, and acceptability studies were flagged during screening, and reviewers will revisit these records to determine if clinical outcomes were published. The final list of included studies, and additional, relevant studies, will be evaluated by the entire review team, along with relevant unaffiliated collaborators. Articles identified through these supplemental methods will proceed through the full screening process.

### Search Strategy

A biomedical librarian (NT) with expertise in systematic review searches developed the search strategy in collaboration with the other members of the study team. A second librarian (Alicia A. Livinski, MPH, MLS) not otherwise affiliated with the project peer-reviewed the search strategy. The review team provided feedback on the search strategy. The search strategy incorporated keywords and controlled vocabulary terms (ie, EMTREE [Embase], MeSH [PubMed], Thesaurus of Psychological Index Terms [PsycNet]) for each concept of interest. The search strategy syntax was adapted for each database searched. Multimedia Appendix 2 shows the PubMed search strategy.

### Study Records

#### Data Management

We used EndNote 20 (Clarivate) to collect and manage the results of the literature search and identify unique records. We used Covidence (Veritas Health Innovations), an internet-based tool for systematic review data management, for selection and data collection. Before selection and data collection, reviewers (LMH, MB-T, CEH, CNR, and ET) were trained in using Covidence.

#### Selection Process

Before each stage of screening (title and abstract, full text), a 20-article trial was conducted with all reviewers to pilot and refine the eligibility criteria to increase reliability. All reviewers are conducting screening using the stated eligibility criteria. For title and abstract screening, articles with missing abstracts automatically advanced to full-text screening. Following the title and abstract screening, the PDFs of the articles that were included were obtained and uploaded into Covidence for full-text screening. All articles are being double-coded for each stage of screening; that is, each record is independently screened by 2 reviewers. Screening discrepancies are being resolved during a group consensus meeting. Interrater reliability is being recorded in Covidence and will be documented in the final report.

#### Data Collection Process

Before data collection, a 15-article trial will be conducted with all reviewers (LMH, MB-T, CEH, CNR, and ET) to increase reliability. All reviewers (LMH, MB-T, CEH, CNR, and ET) will independently extract data items from records included after full-text screening. Each data item from each study record will be extracted in duplicate. Coding discrepancies will be discussed and resolved through dyadic consensus meetings, including the two relevant reviewers for each article. Discrepancies that cannot be resolved during dyadic consensus meetings will be resolved at a group consensus meeting. Data extraction forms will be published.

#### Data Items

Table 1 shows the data items that will be collected.

**Table 1:** Data items for systematic review

Category	Criteria
Article	Authors, title, journal, year.
Sample demographics	Where the study took place, sample size, age, gender, sex, race, and ethnicity.
JITAI^a^ elements	Tailoring variable, decision points, decision rules (including static or adaptive status), intervention options, proximal outcomes (eg, emotion regulation), distal outcomes (eg, depression).
Study	Intervention development framework (eg, National Institutes of Health’s ORBIT^b^ model, MOST^c^), experimental design (eg, MRT^d^, SMART^e^), where the JITAI was developed (eg, university, industry), was the JITAI delivered alongside other support (eg, in-person intervention), was the JITAI delivered alongside sensors or ambulatory devices, types of sensors used, JITAI delivery service (eg, iPhone, personal device), number of intervention days, payment structure (eg, flat fee), user engagement, user compliance (eg, response to prompts or frequency of JITAI use, or both), usability.

^a^JITAI: just-in-time adaptive intervention.

^b^ORBIT: obesity-related behavioral intervention trials.

^c^MOST: multiphase optimization strategy.

^d^MRT: microrandomized trial.

^e^SMART: sequential multiple assignment randomized trial.

### Outcomes and Prioritizations

Our primary outcome of interest is the distal outcome of the JITAI. Here, we focus on JITAIs that target behavioral health outcomes. Examples of potential distal outcomes include affective, substance use, disruptive behavior, eating, trauma-related, personality, psychotic, and neurodevelopmental disorders; psychological symptoms (ie, not meeting criteria for a disorder); pain; and well-being. Multimedia Appendix 2 shows the full list of search terms related to behavioral health. Our secondary outcome of interest is the proximal outcome of the JITAI. All proximal outcomes will be collected. We will calculate effect sizes for both the distal and proximal outcomes.

### Risk of Bias in Individual Studies

Tools appropriate to the study design of individual records will be used to determine risk of bias. For example, Version 2 of the Cochrane risk-of-bias (RoB 2) tool for randomized controlled trials will be used for randomized controlled trials, including SMARTs [42]; Risk Of Bias In Non-Randomized Studies - of Interventions (ROBINS-I) will be used for nonrandomized studies comparing the effects of two or more interventions [43]; and Risk Of Bias In Non-randomized Studies - of Exposure (ROBINS-E) will be used for observational studies [44]. Risk of bias will be evaluated by 2 reviewers for each study. Disputes will be resolved during dyadic consensus meetings, and discrepancies that cannot be resolved during dyadic consensus meetings will be resolved during group consensus meetings.

RoB 2 includes 5 domains: bias due to the randomization process, bias due to deviations from intended interventions, bias due to missing outcome data, bias in the measurement of the outcome, and bias in the selection of the reported result [42]. Each domain will be classified as low risk of bias, some concerns, or high risk of bias. ROBINS-I includes 7 domains: bias due to confounding, bias in the selection of participants into the study, bias in classification of interventions, bias due to deviations from intended interventions, bias due to missing data, bias in the measurement of the outcome, and bias in the selection of the reported result [43]. Each domain will be classified as low, moderate, serious, or critical risk of bias or no information. ROBINS-E includes 7 domains: risk of bias due to confounding, risk of bias arising from the measurement of exposure, risk of bias in the selection of participants into the study, risk of bias due to post-exposure interventions, risk of bias due to missing data, risk of bias arising from the measurement of outcome, and risk of bias in the selection of the reported result [44]. Each domain will be classified as low risk of bias, some concerns, high risk of bias, or very high risk of bias.

### Data Synthesis

Elements (eg, tailoring variables and decision points) of each JITAI will be presented in tables and summarized in the text. Data synthesis will be explored based on outcomes, and a summary of findings table will be presented, as appropriate.

We will categorize studies by design type (eg, randomized controlled trial, observational). If studies in each subgroup are sufficiently homogeneous, meta-analyses will be conducted for the distal and proximal outcomes. Features of the studies collected will inform the selection of the measure of effect size (eg, Cohen's *d* and Hedges' *g*). Subgroup analyses will be considered based on characteristics, including age, gender, type of psychopathology, and type of JITAI. In addition, sensitivity analysis will be carried out, excluding studies of low methodological quality, if necessary.

*I*^2^ statistic will be used to quantify heterogeneity across effect sizes, and *Q* statistic will be used to test heterogeneity reduction through the inclusion of moderators. To address potential heterogeneity, meta-regression will be used to assess clinical characteristics (eg, the severity of symptoms), individual JITAI features (eg, type of emotional regulation strategies used in the intervention), study quality, and demographic characteristics (eg, age, gender, race, and ethnicity) that might influence the effect sizes.

If appropriate, mediation analyses will be considered to investigate whether changes in proximal outcomes mediate the relation between JITAIs and distal outcomes.

### Meta-Biases

Publication bias, resulting from selective publication or reporting, will be investigated through visual inspection of funnel plots. Statistical tests for assessing symmetry (eg, Egger's test) will be explored if 10 or more studies have evaluated the same outcome. Trim and fill analyses will be conducted as necessary.

### Confidence in Cumulative Evidence

The quality of evidence of estimates will be rated using the Grading of Recommendations Assessment, Development, and Evaluation (GRADE) framework [45]. GRADE characterizes the quality of evidence according to publication bias, study limitations, inconsistency, imprecision, and indirectness [17]. Evidence of effectiveness outcomes will be rated from high to low quality by 2 reviewers. Disputes will be resolved during dyadic consensus meetings, and discrepancies that cannot be resolved during dyadic consensus meetings will be resolved during group consensus meetings. Results from GRADE ratings will be included in the summary of findings table.

## Results

The search strategy was developed between July 2023 and November 2023, and the literature search was executed between November 2023 and December 2023. We retrieved 1243 records from our literature search. We excluded 91 duplicate records, leaving 1152 records for the selection process. Of note is that we performed a preliminary literature search for systematic reviews related to this topic and found no review identical to the one proposed. Title and abstract screening began in December 2023, and full-text screening began in May 2024. Data extraction and analyses have not begun.

## Discussion

### Principal Findings

In our systemic review, our objective is to describe the state of the literature on JITAIs for behavioral health outcomes. We expect that there will be sufficient data available to take a qualitative approach, including describing the aforementioned 6 elements for each JITAI. Conditional upon sufficient homogeneity of the included studies, we will also take a quantitative approach to examine the effectiveness of JITAIs in promoting (1) aspects of behavioral health as distal outcomes and (2) proximal outcomes.

### Strengths and Limitations

Our systematic review has numerous strengths, including a comprehensive search strategy, double coding in each stage of screening and data extraction by trained reviewers, assessment of risk of bias of the included studies by reputed tools, evaluation of publication bias, and ratings of quality of evidence of estimates derived from the review.

There are also potential limitations of our work. Primarily, some ambiguity exists in the use of the term “JITAI;” the definition of JITAI has evolved since it was coined in 2015 [19]; the term has been used inconsistently (eg, an intervention with all the characteristics of a JITAI being characterized as a “momentary intervention” or related term) and imprecisely (eg, an intervention labeled as a JITAI despite the lack of an adaptive element); and there is overlap in the definitions of JITAIs and similar intervention frameworks (eg, ecological momentary interventions). As such, we have developed a robust search strategy, including not only the term JITAI but also related terms (eg, ecological momentary intervention, real-time intervention; more details in Multimedia Appendix 2). Furthermore, we developed and implemented detailed eligibility criteria for screening. We also clearly describe the 6 elements that constitute a JITAI in this protocol and will describe each element for each included JITAI in the final report. Accordingly, this systematic review may provide the field with further clarity on the definition and elements of JITAIs. Another limitation is that due to the iterative design process that characterizes JITAIs (and interventions, in general), JITAIs included in this report may not reflect their finalized form. Future scholarship will be needed to capture further JITAI innovations and evaluations. Finally, although we did not limit our search to English language articles, most of our articles are written in English, so the samples and populations represented may be similarly homogeneous. Additional work may be needed to increase the representation of studies published in languages other than English in JITAI effectiveness research.

### Comparison With Previous Work

Previous research has typically evaluated JITAIs as digital health tools for the promotion of physical health [15,16]. While a select few reviews have examined JITAIs for specific psychiatric disorders [13], research has yet to robustly summarize the impact of JITAIs on behavioral health conditions.

### Conclusions

This systematic review will summarize the evidence on the effectiveness of JITAIs in improving distal (ie, behavioral health) and proximal (eg, emotion regulation) outcomes. Results will provide clarity on JITAI definitions and elements, describe the effectiveness of JITAIs for behavioral health, elucidate targeted proximal outcomes, and inform the development of future JITAIs.

## Appendix

Multimedia Appendix 1 PRISMA (Preferred Reporting Items for Systematic Reviews and Meta-Analyses) 2020 checklist.

Multimedia Appendix 2 Search Strategy.

## Abbreviations

GRADE: Grading of Recommendations Assessment, Development and Evaluation
JITAI: just-in-time adaptive intervention
MOST: multiphase optimization strategy
ORBIT: Obesity-Related Behavioral Intervention Trials
PRISMA-P: Preferred Reporting Items for Systematic Reviews and Meta Analyses Protocols
RoB 2: Version 2 of the Cochrane risk-of-bias tool for randomized controlled trials
ROBINS-E: Risk Of Bias In Non-Randomized Studies - of Exposure
ROBINS-I: Risk Of Bias In Non-Randomized Studies - of Interventions
SMART: sequential multiple assignment randomized trial

## Data Availability

The datasets generated during or analyzed during this study will be available in the OSF repository [46].
