# Supplementary material for: Just-In-Time Adaptive Interventions to Promote Behavioral Health: Protocol for a Systematic Review
Source: JMIR Res Protoc. 2025 Feb 11;14:e58917. doi: 10.2196/58917 (PMC11862764; doi:10.2196/58917)
Supplement: Multimedia Appendix 2 [file resprot_v14i1e58917_app2.docx]

Appendix/Supplementary Material

**Title:**  Just-In-Time Adaptive Interventions to Promote Behavioral Health: Protocol for a Systematic Review

**Database:** PubMed/MEDLINE – search strategy
**Platform:** US National Library of Medicine
**Date Searched:** Nov 16, 2023

**Date Limits:** None applied
**Other Limits/Filters:** Human studies

**Notes:** The keywords were searched in the title and abstract fields in PubMed (ie, [Title/Abstract]), and the controlled vocabulary terms are indicated with [MeSH Terms]. Phrases were enclosed in quotation marks to force the searching of the exact terms in order presented. Proximity searching was applied to some phrases to search for those words in the phrase within a specified number of words (ie, [Title/Abstract:~2]). Search results were limited to human studies. To these results, a search strategy to exclude specific publication types was used. No other limits were applied to the search.

| **Set** | **Concept** | **Search Strategy** |
| --- | --- | --- |
| #1 | Just in Time Adaptive Intervention | "just in time adaptive intervention"[Title/Abstract:~2] OR "just in time adaptive interventions"[Title/Abstract:~2] OR "JITAI"[Title/Abstract] OR "JITAIS"[Title/Abstract] OR "just in time support"[Title/Abstract:~2] OR "ecological momentary intervention"[Title/Abstract:~2] OR "ecological momentary interventions"[Title/Abstract:~2] OR "intelligent real-time therapy"[Title/Abstract:~2] OR "intelligent real-time therapies"[Title/Abstract:~2] OR "dynamic tailoring"[Title/Abstract] OR "dynamically tailor"[Title/Abstract:~2] OR "dynamically tailored"[Title/Abstract:~2] OR "Addiction Comprehensive Health Enhancement Support System"[Title/Abstract] OR "Comprehensive Health Enhancement Support System"[Title/Abstract] OR "adaptive monitoring"[Title/Abstract:~2] OR "momentary intervention"[Title/Abstract:~2] OR "momentary interventions"[Title/Abstract:~2] OR "real time intervention"[Title/Abstract:~2] OR "real time interventions"[Title/Abstract:~2] OR "personal health intervention toolkit"[Title/Abstract:~2] OR "personal health intervention toolkits"[Title/Abstract:~2] OR "PHIT"[Title/Abstract] |
| #2 | Substance Use | "substance use"[Title/Abstract:~2] OR "substance usage"[Title/Abstract:~2] OR "substance abuse"[Title/Abstract:~2] OR "substance misuse"[Title/Abstract:~2] OR "substance misuse"[Title/Abstract:~2] OR "substance addiction"[Title/Abstract:~2] OR "substance addicted"[Title/Abstract:~2] OR "substance dependence"[Title/Abstract:~2] OR "substance dependent"[Title/Abstract:~2] OR "drug addiction"[Title/Abstract:~2] OR "drug habituation"[Title/Abstract:~2] OR "drug habit"[Title/Abstract:~2] OR "drug habits"[Title/Abstract:~2] OR "drug abuse"[Title/Abstract:~2] OR "drug dependence"[Title/Abstract:~2] OR "drug use"[Title/Abstract:~2] OR "drug misuse"[Title/Abstract:~2] OR "drug overdose"[Title/Abstract:~2] OR "drug addicted"[Title/Abstract:~2] OR "drug habituation"[Title/Abstract:~2] OR "drug abusing"[Title/Abstract:~2] OR "drug dependent"[Title/Abstract:~2] OR "drug using"[Title/Abstract:~2] OR "drug misusing"[Title/Abstract:~2] OR "opioid addiction"[Title/Abstract:~2] OR "opioid abuse"[Title/Abstract:~2] OR "opioid dependence"[Title/Abstract:~2] OR "opioid use"[Title/Abstract:~2] OR "opiate addiction"[Title/Abstract:~2] OR "opiate abuse"[Title/Abstract:~2] OR "opiate dependence"[Title/Abstract:~2] OR "opiate use"[Title/Abstract:~2] OR "opiate overdose"[Title/Abstract:~2] OR "opioid addicted"[Title/Abstract:~2] OR "opioid abusing"[Title/Abstract:~2] OR "opioid dependent"[Title/Abstract:~2] OR "opioid usage"[Title/Abstract:~2] OR "opiate abusing"[Title/Abstract:~2] OR "opiate dependent"[Title/Abstract:~2] OR "heroin dependence"[Title/Abstract:~2] OR "morphine dependence"[Title/Abstract:~2] OR "cocaine addiction"[Title/Abstract:~2] OR "cocaine abuse"[Title/Abstract:~2] OR "cocaine dependence"[Title/Abstract:~2] OR "cocaine use"[Title/Abstract:~2] OR "cocaine overdose"[Title/Abstract:~2] OR "amphetamine addiction"[Title/Abstract:~2] OR "amphetamine abuse"[Title/Abstract:~2] OR "amphetamine dependence"[Title/Abstract] OR "amphetamine use"[Title/Abstract:~2] OR "amphetamine overdose"[Title/Abstract:~2] OR "inhalant addiction"[Title/Abstract:~2] OR "inhalant abuse"[Title/Abstract:~2] OR "inhalant dependence"[Title/Abstract:~2] OR "inhalant use"[Title/Abstract:~2] OR "methamphetamine addiction"[Title/Abstract] OR "methamphetamine abuse"[Title/Abstract:~2] OR "methamphetamine dependence"[Title/Abstract] OR "methamphetamine use"[Title/Abstract:~2] OR "methamphetamine overdose"[Title/Abstract:~2] OR "fentanyl addiction"[Title/Abstract:~2] OR "fentanyl abuse"[Title/Abstract:~2] OR "fentanyl use"[Title/Abstract:~2] OR "fentanyl overdose"[Title/Abstract:~2] OR "heroin dependent"[Title/Abstract:~2] OR "morphine dependent"[Title/Abstract:~2] OR "cocaine addicted"[Title/Abstract:~2] OR "cocaine abusing"[Title/Abstract:~2] OR "cocaine dependent"[Title/Abstract:~2] OR "cocaine usage"[Title/Abstract:~2] OR "cocaine overdose"[Title/Abstract:~2] OR "amphetamine addicted"[Title/Abstract:~2] OR "amphetamine abusing"[Title/Abstract:~2] OR "amphetamine dependent"[Title/Abstract:~2] OR "amphetamine usage"[Title/Abstract:~2] OR "inhalant addicted"[Title/Abstract:~2] OR "inhalant abusing"[Title/Abstract:~2] OR "inhalant dependent"[Title/Abstract:~2] OR "inhalant usage"[Title/Abstract:~2] OR "methamphetamine addicted"[Title/Abstract] OR "methamphetamine abusing"[Title/Abstract:~2] OR "methamphetamine dependent"[Title/Abstract] OR "methamphetamine usage"[Title/Abstract:~2] OR "methamphetamine overdosing"[Title/Abstract:~2] OR "fentanyl addicted"[Title/Abstract:~2] OR "fentanyl abusing"[Title/Abstract:~2] OR "fentanyl usage"[Title/Abstract:~2] OR "fentanyl overdosing"[Title/Abstract:~2] OR "fentanyl dependent"[Title/Abstract:~2] OR "fentanyl dependence"[Title/Abstract:~2] OR "substance related disorders"[MeSH Terms] OR "opioid related disorders"[MeSH Terms] OR "illicit drugs"[MeSH Terms] OR "club drug*"[Title/Abstract] OR "illegal drug*"[Title/Abstract] OR "recreational drug*"[Title/Abstract] OR "street drug*"[Title/Abstract] OR ((substance[tiab] OR drug*[tiab] OR opioid*[tiab] OR opiate*[tiab] OR cocaine[tiab] OR amphetamine*[tiab] OR inhalan*[tiab] OR methamphetamine*[tiab] OR fentanyl[tiab] OR narcotic*[tiab]) AND ( use[tiab] OR usage[tiab] OR abus*[tiab] OR dependen*[tiab] OR misuse*[tiab] OR overdos*[tiab] OR addict*[tiab] OR habituate*[tiab])) |
| #3 | Alcohol Use | "alcoholic intoxication"[MeSH Terms] OR "binge drinking"[MeSH Terms] OR "drinking behavior"[MeSH Terms] OR "alcoholism"[Title/Abstract] OR "alcoholism"[MeSH Terms] OR "alcoholic*"[Title/Abstract] OR "alcohol abuse"[Title/Abstract:~2] OR “alcohol abusing”[Title/Abstract:~2] OR "alcohol addiction"[Title/Abstract:~2] OR “alcohol addicted”[Title/Abstract:~2] OR "alcohol depend*"[Title/Abstract] OR “alcohol dependent”[Title/Abstract:~2] OR "alcohol dependence"[Title/Abstract:~2] OR "alcohol use disorder"[Title/Abstract:~2] OR "alcohol use disorders"[Title/Abstract:~2] OR "drunkenness"[Title/Abstract] OR "binge drink*"[Title/Abstract] OR "alcohol intoxicat*"[Title/Abstract] OR "alcohol use"[Title/Abstract:~2] OR "alcohol useage"[Title/Abstract:~2] OR “alcohol using”[Title/Abstract:~2] OR "alcohol misuse"[Title/Abstract:~2] OR “alcohol misusing”[Title/Abstract:~2] OR "drinking episode"[Title/Abstract:~2] OR "drinking episodes"[Title/Abstract:~2] OR "alcohol drink"[Title/Abstract:~2] OR "alcohol drinking"[Title/Abstract:~2] OR "drunken driving"[Title/Abstract:~2] "driving drunk"[Title/Abstract:~2] OR "heavy drink*"[Title/Abstract] OR "college drink"[Title/Abstract] OR "college drinking"[Title/Abstract:~2] OR "underage drink*"[Title/Abstract] OR “social drink”[Title/Abstract:~2] OR “drinking socially”[Title/Abstract:~2] |
| #4 | Tobacco Use | "tobacco use"[Title/Abstract:~2] OR "tobacco addict"[Title/Abstract:~2] OR "tobacco dependence"[Title/Abstract:~2] OR "tobacco dependent"[Title/Abstract:~2] OR "tobacco use cessation"[Title/Abstract] OR "nicotine addict"[Title/Abstract:~2] OR "nicotine dependence"[Title/Abstract:~2] OR "nicotine dependent"[Title/Abstract:~2] OR "nicotine use"[Title/Abstract:~2] OR "smoking"[Title/Abstract] OR "nicotine dependence"[Title/Abstract:~2] OR "smoking cessation"[MeSH Terms] OR "smoking reduction"[MeSH Terms] OR "smoker*"[Title/Abstract] OR "vaping"[Title/Abstract] OR "e-cigarette addiction"[Title/Abstract:~2] OR "electronic cigarette dependence"[Title/Abstract:~2] OR "electronic cigarette use"[Title/Abstract:~2] OR "electronic cigarette dependence"[Title/Abstract:~2] OR "electronic cigarette use"[Title/Abstract:~2] OR "e cigarette*"[Title/Abstract] OR "electronic cigarette*"[Title/Abstract] OR "vaping"[MeSH Terms] OR "tobacco use cessation"[MeSH Terms] OR "tobacco use"[MeSH Terms] OR "tobacco, smokeless"[MeSH Terms] OR "snuff"[Title/Abstract] OR "chewing tobacco"[Title/Abstract] OR "smokeless tobacco"[Title/Abstract] |
| #5 | Pain, Mental Health, Emotion Regulation | pain[mh] OR pain[tiab] OR painful[tiab] OR stress, psychological[mh] OR "psychological stress"[tiab] OR stress disorders, post-traumatic[mh] OR "post traumatic stress disorder*"[tiab] OR "posttraumatic stress disorder*"[tiab] OR "stress management"[tiab] OR “managing stress”[Title/Abstract:~2] OR ptsd[tiab] OR "emotional reactivity"[tiab] OR "emotionally reactive"[tiab] OR emotions[mh] OR emotion*[tiab] OR emotional regulation[mh] OR "emotional regulation"[tiab] OR “emotion regulation”[Title/Abstract:~2] OR “emotion regulating”[Title/Abstract:~2] OR “emotions regulating”[Title/Abstract:~2] OR "emotional expression"[tiab] OR "psychological distress*"[tiab] OR "Psychological Distress"[majr] OR "anxiety"[tiab] OR "mental health"[tiab] OR "mental disorders"[tiab] OR adaptation, psychological[mh] |
| #6 | Additional Disorders | ("somatoform"[Title/Abstract] OR "Somatoform Disorders"[MeSH Major Topic] OR "eating disorder*"[Title/Abstract] OR "Feeding and Eating Disorders"[MeSH Major Topic] OR "eating patho*"[Title/Abstract] OR "obsessive compulsive disorder*"[Title/Abstract] OR "Obsessive-Compulsive Disorder"[MeSH Major Topic:noexp] OR "personality disorder*"[Title/Abstract] OR "Personality Disorders"[MeSH Major Topic] OR "self-harm"[Title/Abstract] OR "self-harm"[Title/Abstract] OR "Self-Injurious Behavior"[MeSH Major Topic] OR "self injur*"[Title/Abstract] OR "self injur*"[Title/Abstract] OR "sexual problem*"[Title/Abstract] OR "anorex*"[Title/Abstract] OR "anorexia"[MeSH Major Topic] OR "anxieties"[Title/Abstract] OR "anxious*"[Title/Abstract] OR "anxiety"[MeSH Major Topic] OR "Anxiety Disorders"[MeSH Major Topic] OR "binge eat*"[Title/Abstract] OR "Binge-Eating Disorder"[MeSH Major Topic] OR "bipolar"[Title/Abstract] OR "Bipolar Disorder"[MeSH Major Topic] OR "bulim*"[Title/Abstract] OR "bulimia"[MeSH Major Topic] OR "depressi*"[Title/Abstract] OR ("Depressive Disorder"[MeSH Major Topic] OR "depression"[MeSH Major Topic]) OR "Depressive Disorder"[MeSH Major Topic] OR "dysthymia*"[Title/Abstract] OR "Dysthymic Disorder"[MeSH Major Topic] OR "fear"[Title/Abstract] OR "fear"[MeSH Major Topic] OR "hypomani*"[Title/Abstract] OR "mania"[MeSH Major Topic] OR "internaliz*"[Title/Abstract] OR "mania*"[Title/Abstract] OR "manic"[Title/Abstract] OR "panic"[Title/Abstract] OR "panics"[Title/Abstract] OR "panicked"[Title/Abstract] OR "panic"[MeSH Major Topic] OR "Panic Disorder"[MeSH Major Topic] OR "phobi*"[Title/Abstract] OR "Phobic Disorders"[MeSH Major Topic] OR "suicid*"[Title/Abstract] OR "suicide"[MeSH Major Topic] OR "trauma*"[Title/Abstract] OR "thought disorder*"[Title/Abstract] OR "paranoi*"[Title/Abstract] OR "Paranoid Disorders"[MeSH Major Topic] OR "psychosis"[Title/Abstract] OR "Psychotic Disorders"[MeSH Major Topic] OR "schizo*"[Title/Abstract] OR "schizophrenia"[MeSH Major Topic] OR "anger control"[Title/Abstract] OR "attention deficit*"[Title/Abstract] OR "Attention Deficit and Disruptive Behavior Disorders"[MeSH Major Topic] OR "attention problem*"[Title/Abstract] OR "behavior problem*"[Title/Abstract] OR "Problem Behavior"[MeSH Major Topic] OR "behaviour problem*"[Title/Abstract] OR "behavioral development*"[Title/Abstract] OR "behavioural development*"[Title/Abstract] OR "behavioral outcome*"[Title/Abstract] OR "behavioural outcome*"[Title/Abstract] OR "behavioral well-being"[Title/Abstract] OR "behavioural well-being"[Title/Abstract] OR "behavioral wellbeing"[Title/Abstract] OR "behavioural wellbeing"[Title/Abstract] OR "disruptive behavior*"[Title/Abstract] OR "disruptive behaviour*"[Title/Abstract] OR "intermittent explosive disorder*"[Title/Abstract] OR "disruptive, impulse control, and conduct disorders"[MeSH Major Topic] OR "oppositional defiant disorder*"[Title/Abstract] OR "aggress*"[Title/Abstract] OR "aggression"[MeSH Major Topic] OR "antagonistic"[Title/Abstract] OR "antisocial"[Title/Abstract] OR "Antisocial Personality Disorder"[MeSH Major Topic] OR "conduct disorder*"[Title/Abstract] OR "Conduct Disorder"[MeSH Major Topic] OR "disinhibit*"[Title/Abstract] OR "externaliz*"[Title/Abstract] OR "hyperactiv*"[Title/Abstract] OR "hyperkine*"[Title/Abstract] OR "impulsiv*"[Title/Abstract] OR "Impulsive Behavior"[MeSH Major Topic] OR "inattenti*"[Title/Abstract] OR "irritab*"[Title/Abstract] OR "sociopath*"[Title/Abstract] OR "communication disorder*"[Title/Abstract] OR "Communication Disorders"[MeSH Major Topic] OR "coordination disorder*"[Title/Abstract] OR "developmental delay*"[Title/Abstract] OR "developmental disorder*"[Title/Abstract] OR "Developmental Disabilities"[MeSH Major Topic] OR "fluency disorder*"[Title/Abstract] OR "intellectual disab*"[Title/Abstract] OR "Intellectual Disability"[MeSH Major Topic] OR "language disorder*"[Title/Abstract] OR "Language Disorders"[MeSH Major Topic] OR "Language Development Disorders"[MeSH Major Topic] OR "learning disorder*"[Title/Abstract] OR "Learning Disabilities"[MeSH Major Topic] OR "motor disorder*"[Title/Abstract] OR "Motor Disorders"[MeSH Major Topic] OR "movement disorder*"[Title/Abstract] OR "Movement Disorders"[MeSH Major Topic] OR "speech sound disorder*"[Title/Abstract] OR "Speech Sound Disorder"[MeSH Major Topic] OR "Speech Disorders"[MeSH Major Topic] OR "autis*"[Title/Abstract] OR "Autism Spectrum Disorder"[MeSH Major Topic] OR "Autistic Disorder"[MeSH Major Topic] OR "Asperger Syndrome"[MeSH Major Topic] OR "neurodevelopmental disorder*"[Title/Abstract] OR "Neurodevelopmental Disorders"[MeSH Major Topic] OR "stutter*"[Title/Abstract] OR "stuttering"[MeSH Major Topic] OR "tic"[Title/Abstract] OR "tics"[Title/Abstract] OR "tics"[MeSH Major Topic] OR "tourette*"[Title/Abstract] OR "Tourette Syndrome"[MeSH Major Topic] OR "mental disorder*"[Title/Abstract] OR "Mental Disorders"[MeSH Major Topic:noexp] OR "mental illness*"[Title/Abstract] OR "psychological distress*"[Title/Abstract] OR "Psychological Distress"[MeSH Major Topic] OR "psychological impair*"[Title/Abstract] OR "psychological symptom*"[Title/Abstract] OR "psychopatho*"[Title/Abstract] OR "psychopathology"[MeSH Major Topic]) |
| #7 | Coping/Adaptation | Coping[Title/Abstract] OR adaptation, psychological[mh] OR "psychological adaptation"[Title/Abstract:~2] OR acceptance[Title/Abstract] OR "adaptive behavior"[Title/Abstract:~2] OR “healthy adaptation”[Title/Abstract:~2] OR “positive adaptation”[Title/Abstract:~2] OR "behavioral avoidance"[Title/Abstract:~2] OR "cognitive reappraisal"[Title/Abstract:~2] OR denial[Title/Abstract] OR denial, psychological[mh] OR disengag*[Title/Abstract] OR "positive thinking"[Title/Abstract:~2] OR "thinking positively"[Title/Abstract] OR "positive attitude"[Title/Abstract:~2] OR optimism[mh] OR "diaphragmatic breathing"[Title/Abstract] OR "diaphragm breathing"[Title/Abstract:~2] OR “deep breathing”[Title/Abstract:~2] OR “breathing deeply”[Title/Abstract:~2] OR distraction[Title/Abstract] OR humor*[Title/Abstract] OR humour*[Title/Abstract] OR laughter therapy[mh] OR laughter[Title/Abstract] OR "expressive suppression"[Title/Abstract:~2] OR problem solving[mh] OR "problem solving"[Title/Abstract:~2] OR "progressive muscle relaxation"[Title/Abstract:~2] OR autogenic training[mh] OR "progressive relaxation"[Title/Abstract:~2] OR reframing[Title/Abstract] OR cognitive restructuring[mh] OR resignation[Title/Abstract] OR help seeking behavior[mh] OR "support seeking"[tiab:~2] OR "social skills"[Title/Abstract:~2] OR social skills[mh] OR "thought suppression"[Title/Abstract:~2] OR “thought suppressing”[Title/Abstract:~2] OR repression, psychology[mh] OR engage*[Title/Abstract] |
| #8 |  | #1 AND (#2 OR #3 OR #4 OR #5 OR #6 OR #7) |
| #9 | Filters Used: Humans, Publication Type Exclusions | #8 NOT ("Animals"[MeSH Terms] NOT ("Animals"[MeSH Terms] AND "Humans"[MeSH Terms]))) NOT ("letter"[Publication Type] OR "retracted publication"[Publication Type] OR "retraction of publication"[Publication Type] OR "retraction of publication"[Title/Abstract] OR "retraction notice"[Title] OR "retracted publication"[Title] OR "conference abstract*"[Title/Abstract] OR "conference proceeding*"[Title/Abstract] OR "conference paper*"[Title/Abstract]) |

**ABOUT PUBMED**

This search strategy was created to search the PubMed database. [PubMed](https://pubmed.ncbi.nlm.nih.gov) is a free resource supporting the search and retrieval of biomedical and life sciences literature. The PubMed database contains more than 37 million citations and abstracts of biomedical literature and is considered one of the required databases to search in a systematic review/meta-analysis. PubMed was developed and is maintained by the National Center for Biotechnology Information (NCBI), at the U.S. National Library of Medicine (NLM), located at the National Institutes of Health (NIH).

**SEARCHING**

To search, we began by identifying the key concepts from the research question for this systematic review. We have identified key concepts in this review and included them separate rows in the search documentation table. Each concept’s row includes a set of search terms describing these concepts.

In a systematic review we try to search as broadly as possible to identify as many records as we can related to the topic of the review. Hence, there is a large number of search terms describing the concepts. We try to balance specificity with sensitivity. A sensitive search attempts to retrieve all relevant documents by using a broad search. A specific search attempts to retrieve only relevant documents in a small precise search. In a systematic review we try to reach a happy medium between sensitivity and specificity.

**SEARCH SYNTAX EXPLANATION**

The explanation below is specific to PubMed; however, the general approaches must be adapted to search other databases due to variations in syntax, field names, and other search features.

The search syntax for this PubMed search used a combination of fields that can be found in a PubMed record. We mainly used the Medical Subject Heading [MeSH] field and the [Title/Abstract] field. The name of the fields to be searched are enclosed in square brackets (eg, [MeSH], [Title]). We use specific fields to control how PubMed processes our search.

Some of our search terms are searched as phrases. Phrases are enclosed in double quotation marks which specify that the word combinations be searched together as a phrase.

The search also used proximity searching which specifies the number of words that can appear between the words in the phrase.

We also used a truncator symbol, the asterisk (*) which searches on the root word and any other variations of the word containing the root word.

Please see examples of the search syntax we used below. Please note that this syntax can be applied to each concept.

**SEARCH SYNTAX EXAMPLES:**

Below we provide examples of the search syntax contained in the PubMed search strategy we used for this systematic review.

- Terms in a specific field by can be searched by including a search field tag after the term. For example, the search field tag **[Title/Abstract]** used in PubMed to search for words included in a citation's title, collection title, abstract, other abstract and author keywords. EXAMPLE FROM SEARCH STRATEGY: "dynamic tailoring"[Title/Abstract]
- **MeSH** is the abbreviation for Medical Subject Headings and is a thesaurus containing controlled and hierarchically organized vocabulary produced by the National Library of Medicine. MeSH terms in the thesaurus can be used for searching for biomedical and health-related information in PubMed and are used to describe the subject of each journal article in PubMed. EXAMPLE FROM SEARCH STRATEGY: "Substance Related Disorders"[MeSH Terms]
- **Double Quotation Marks** are used to search two or more words together as a phrase. EXAMPLE FROM SEARCH STRATEGY: "just in time adaptive intervention"
- **Asterisk (***) is a wildcard symbol to substitute for 0 or more characters in a term or phrase. The asterisk is placed at the root of the word to find variations (eg, dependen*[Title/Abstract] finds PubMed records with the words dependent, dependents, dependency, dependencies, or dependence in the title or the abstract). EXAMPLE FROM SEARCH STRATEGY: dependen*[TITLE/Abstract]
- **Proximity Searching** is used to search for multiple terms appearing in any order within a specified distance of one another in the [Title] or the [Title/Abstract] fields of a PubMed record. A proximity number of 2 was chosen for this search to specify that no more than 2 words can appear between the search terms. A low number such as 2 creates a narrower more precise search. Results will include the quoted terms in any order. EXAMPLE FROM SEARCH STRATEGY: "drug misuse"[Title/Abstract:~2]

**COMBINING ALL THE SEARCH TERMS TOGETHER**

For each ***single*** concept, a combination of single words, phrases, and MeSH terms describing that single concept are linked together with the connecting word (commonly referred to as a Boolean operator) “OR”. OR retrieves results that include at least one of the search terms within that single concept. EXAMPLE FROM THE SEARCH: (#2 OR #3 OR #4 OR #5 OR #6 OR #7)

Another Boolean operator, “AND”, is used to link together each single concept to be searched. The word AND specifies that the search retrieves results from each concept. From the example, at least 1 word from the JITAI search concept (ie, #1) AND at least 1 term from any of the other concepts (ie, #2, 3, 4, 5, 6, 7) must be present in any result retrieved from the search in PubMed. EXAMPLE FROM THE SEARCH: #1 AND (#2 OR #3 OR #4 OR #5 OR #6 OR #7)

Note that PubMed processes searches in a left to right sequence. We used parentheses to group or nest words describing each concept together. Parentheses are required to allow PubMed to process the words for each concept as a unit and then be incorporated into the overall search.

The final concepts in the search are publication types such as letters, editorials, etc. including retracted studies that are *to be excluded from the final search results*.

We use the Boolean operator, “NOT” to filter out or exclude what we did not want included in the search results. In this example, we wanted to exclude animal studies and specific publication types (eg, letters, retractions, conference proceedings). EXAMPLE FROM THE SEARCH: NOT ("Animals"[MeSH Terms] NOT ("Animals"[MeSH Terms] AND "Humans"[MeSH Terms]))) NOT ("letter"[Publication Type] OR "retracted publication"[Publication Type] OR "retraction of publication"[Publication Type] OR "retraction of publication"[Title/Abstract] OR "retraction notice"[Title] OR "retracted publication"[Title] OR "conference abstract*"[Title/Abstract] OR "conference proceeding*"[Title/ Abstract] OR "conference paper*"[Title/Abstract])
